# TIMELESS promotes glioma stemness and malignancy through JAK-STAT3 pathway

**DOI:** 10.1016/j.jbc.2026.113447

**Published:** 2026-08-13

**Authors:** Yuichiro Kai, Akito Tsuruta, Takuto Inoki, Tomoaki Yamauchi, Shigehiro Ohdo, Satoru Koyanagi

**Affiliations:** 1 Department of Pharmaceutics, Faculty of Pharmaceutical Sciences, Kyushu University, Fukuoka, Japan; 2 Department of Clinical Pharmacokinetics, Faculty of Pharmaceutical Sciences, Kyushu University, Fukuoka, Japan

**Keywords:** glioma, clock gene, TIMELESS, cancer stem cells, JAK-STAT3

## Abstract

Gliomas are the most common primary tumors of the central nervous system. Among them, glioblastoma (GBM), a World Health Organization (WHO) grade 4 glioma, is the most aggressive form and remains one of the most lethal human cancers. Its poor prognosis is largely attributable to rapid progression, frequent recurrence, and profound therapeutic resistance, all of which are closely associated with glioma stem cells (GSCs). Although several signaling pathways sustaining GSC properties have been identified, upstream regulators that coordinate these pathways remain incompletely understood. TIMELESS, originally identified as a component of the circadian clock, has recently been implicated in the regulation of brain function and is aberrantly overexpressed in multiple cancer types; however, its role in glioma malignancy has not been defined. Here, we demonstrate that *TIMELESS* mRNA expression increases with glioma grade and is associated with poor prognosis across multiple glioma cohorts. Genetic depletion of *Timeless* suppressed tumor aggressiveness and prolonged survival in an orthotopic mouse glioma model by attenuating GSC properties. Mechanistically, TIMELESS enhanced stemness of glioma by upregulating Janus kinases (JAK) expression and promoting phosphorylation of signal transducer and activator of transcription 3 (STAT3). These effects were conserved in human GBM cells, supporting the relevance of TIMELESS-mediated signaling across species. Together, our findings uncover an unexpected, clock-independent function of TIMELESS in sustaining glioma stemness and malignancy and highlight the TIMELESS-JAK-STAT3 axis as a noncanonical mechanism that operates beyond the traditional circadian clockwork in treatment-resistant glioma.

Gliomas are the most frequently diagnosed primary tumors of the central nervous system and are classified into different grades based on their histological and molecular characteristics. Among them, glioblastoma (GBM), classified as a World Health Organization (WHO) grade 4 glioma, is the most aggressive form and remains one of the most lethal human cancers (1, 2). Despite maximal surgical resection followed by radiotherapy and temozolomide (TMZ) chemotherapy, the median overall survival of GBM patients is only 12 to 15 months, and long-term survival remains rare (1, 2). Tumor recurrence is almost inevitable, highlighting a fundamental limitation of current therapeutic strategies.

A major contributor to treatment resistance and tumor recurrence in GBM is the persistence of glioma stem cell (GSC)-like populations. These cells possess self-renewal capacity, multilineage differentiation potential, and strong tumor-initiating activity, and are highly resistant to conventional therapies (3, 4). Accumulating evidence indicates that GSC maintenance is governed by a tightly regulated transcriptional network involving stemness-associated factors such as OCT4, SOX2, KLF4, and c-MYC, as well as oncogenic signaling pathways, most notably the JAK-STAT3 axis (5, 6, 7, 8). Persistent STAT3 activation promotes GSC survival and reinforces stemness programs, positioning this pathway as a central regulator of glioma malignancy (6, 7, 8).

Notably, most studies on GSC regulation have implicitly assumed that stemness-associated signaling operates in a time-independent manner. However, emerging evidence suggests that stem cell function and tumor biology are subject to temporal regulation by the circadian clock. The circadian system orchestrates daily rhythms in cellular metabolism, DNA repair, and cell cycle progression, processes that are intimately linked to cancer development (9, 10, 11). Dysregulation of circadian clock genes has been associated with increased cancer risk and poor prognosis (12, 13, 14), and recent studies have begun to implicate core clock components in the maintenance of glioma stemness (15). These findings raise the possibility that temporal regulatory mechanisms may represent an unrecognized layer of control over GSC properties.

TIMELESS was originally identified as a core component of the circadian clock machinery in *Drosophila*, functioning within the transcriptional–translational feedback loop that generates circadian rhythms (16). In mammals, TIMELESS has been implicated in DNA replication, cell cycle regulation, and maintenance of genomic stability, and its dysregulation has been linked to tumor progression in several cancer types (17, 18). Notably, analyses of publicly available cancer transcriptome datasets reveal that TIMELESS expression is broadly elevated across multiple malignancies and progressively increases with glioma grade. This observation prompted us to investigate whether TIMELESS contributes to glioma malignancy through regulation of GSC properties. Despite its emerging roles in cancer biology, the functional significance of TIMELESS in glioma stemness and tumor progression remains largely unexplored.

In this study, we found that *TIMELESS* expression increased with glioma grade and was associated with poor patient prognosis. Genetic depletion of *TIMELESS* suppressed tumor aggressiveness and prolonged survival in an orthotopic mouse glioma model by attenuating GSC properties. Mechanistically, *Timeless* depletion downregulated JAK expression and reduced STAT3 phosphorylation, thereby disrupting a core stemness-maintaining pathway. Importantly, these effects were conserved in both mouse glioma and human GBM cells. Our findings uncover an unexpected role of the circadian-associated factor TIMELESS as a critical regulator of glioma stemness and malignancy and identify the TIMELESS-JAK-STAT3 axis as a potential therapeutic target for overcoming stemness-driven treatment resistance in malignant glioma.

## Results

### Association between *TIMELESS* expression and glioma grade and patient prognosis

**Figure 1:**
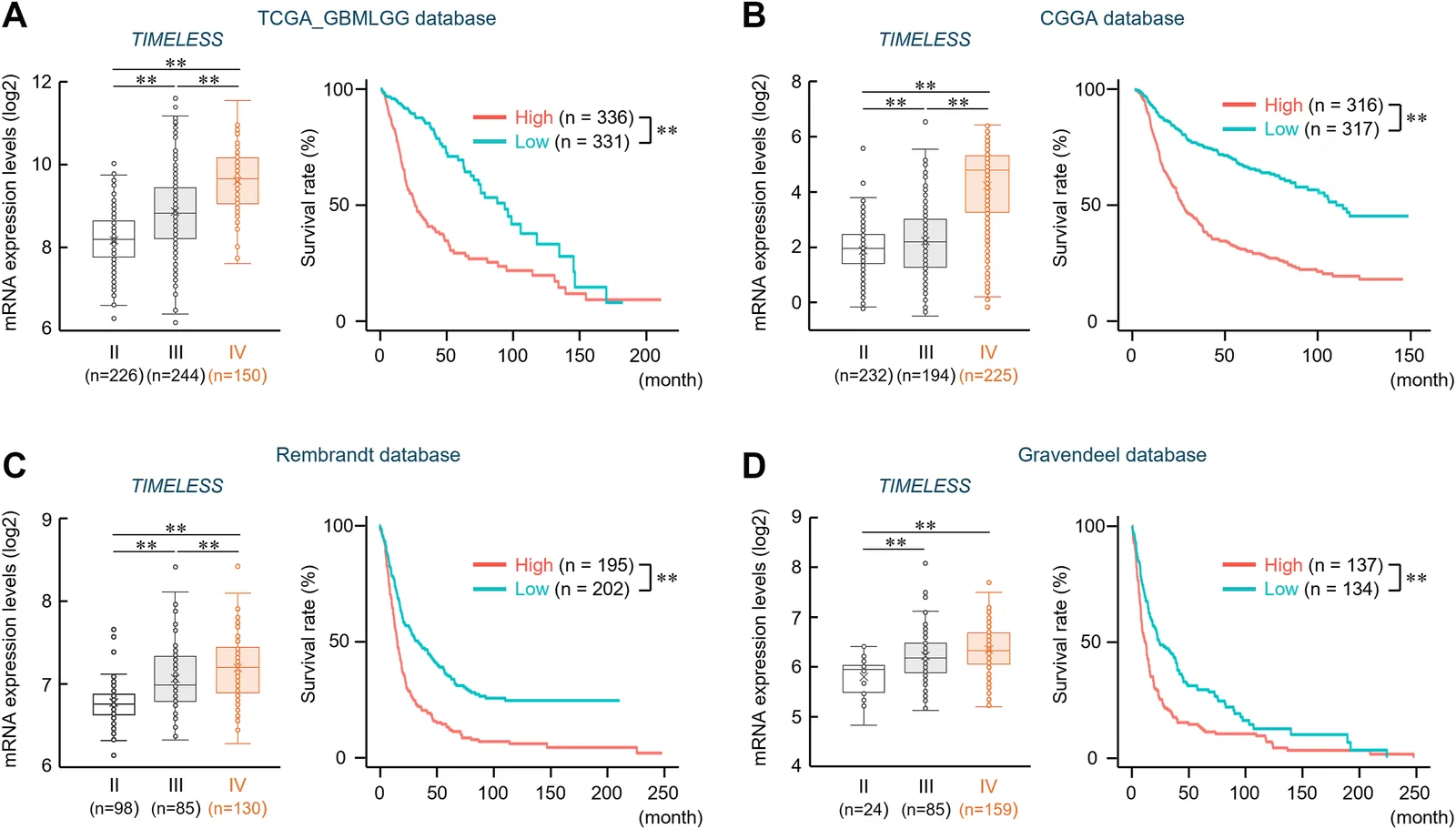
**Database analysis of association between *TIMELESS* expression and patient prognosis.** Glioma patient data were obtained from the TCGA_GBMLGG (*A*), the CGGA (*B*), the Rembrandt (*C*), and the Gravendeel (*D*) datasets and analyzed using GlioVis platform. The dot plots shown on the left in each panel indicate *TIMELESS* mRNA expression levels across glioma grades. ∗∗*p* < 0.01; significant differences between the indicated groups (*F*_2,617_ = 139.918, *p* < 0.001 for the TCGA_GBMLGG data, *F*_2,648_ = 49.497, *p* < 0.001 for the CGGA data, *F*_2,310_ = 40.085, *p* < 0.001 for the Rembrandt data, *F*_2,265_ = 13.932, *p* < 0.001 for the Gravendeel data, ANOVA with Tukey–Kramer’s *post hoc* test). Kaplan–Meier survival curves shown on right graph in each panels depict overall survival of glioma patients stratified by *TIMELESS* mRNA expression levels (high, > median; low, ≤ median). ∗∗*p* < 0.01; significant difference between the high- and low-expression groups (LogRank Holm-Sidak test). TCGA, the cancer genome atlas; GBM, glioblastoma; CGGA, chinese glioma genome atlas.

To investigate the relationship between TIMELESS expression and glioma malignancy, we analyzed *TIMELESS* mRNA levels in glioma tissues using Gliovis (19), a portal for visualization and analysis of brain tumor expression datasets (https://gliovis.bioinfo.cnio.es/). In the TCGA Glioblastoma Multiforme and Low-Grade Glioma (GBMLGG) cohort, *TIMELESS* mRNA expression increased with glioma grade (Fig. 1*A*, left). Kaplan–Meier survival analysis further revealed that patients with high *TIMELESS* expression had significantly shorter survival than those with low expression (Fig. 1*A*, right). We validated these findings in independent cohorts, including the Chinese Glioma Genome Atlas (CGGA), Rembrandt, and the Gravendeel datasets. Across all cohorts, *TIMELESS* expression correlated with glioma grade, and higher *TIMELESS* expression was consistently associated with shorter patient survival (Fig. 1, *B*–*D*). Collectively, these results, consistent with previous reports (20), indicate that elevated TIMELESS expression is associated with glioma malignancy and poorer patient prognosis across grades.

### TIMELESS promotes glioma malignancy in an orthotopic mouse model

**Figure 2:**
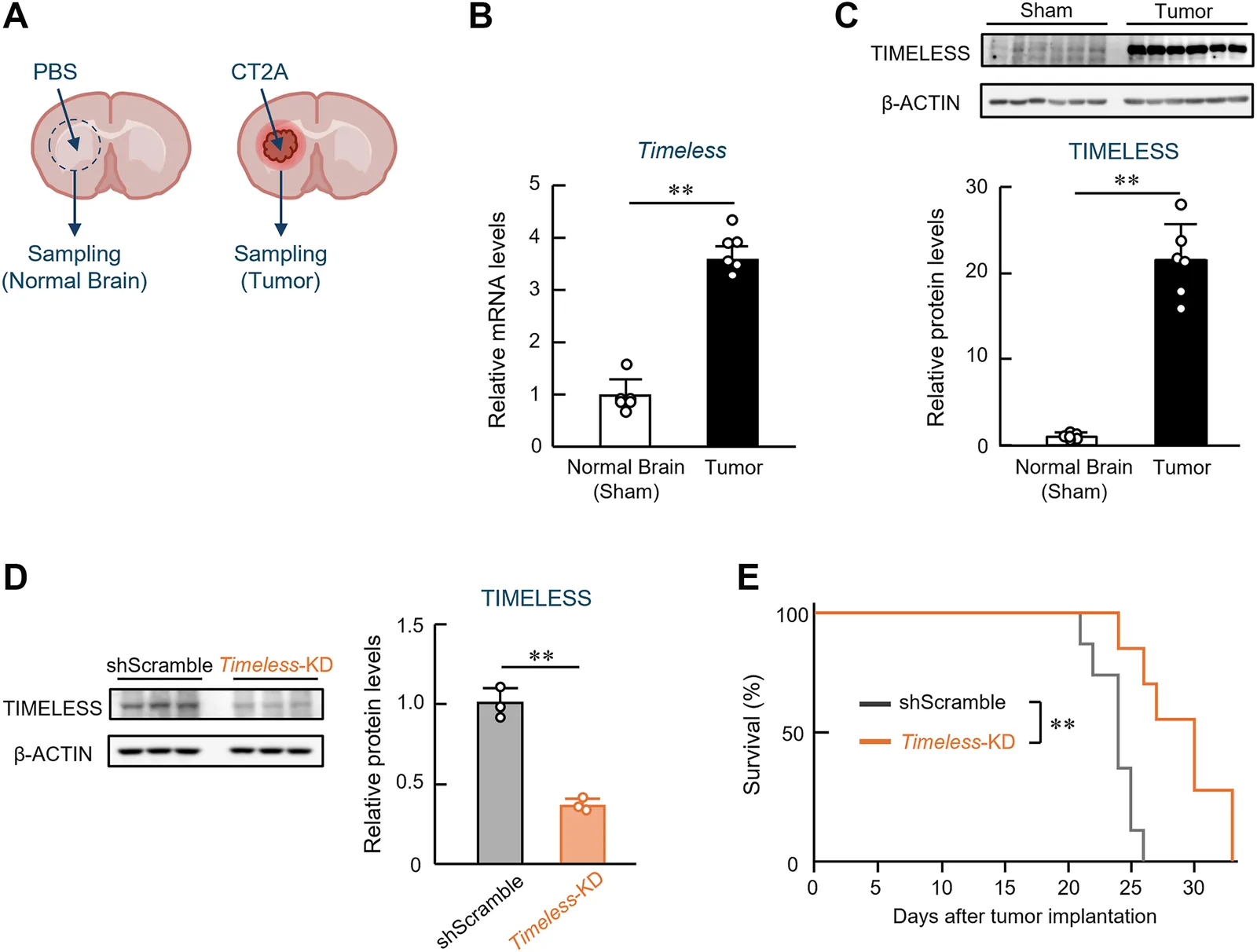
**TIMELESS promotes mouse glioma aggressiveness.***A*, schematic illustration of the orthotopic CT2A glioma transplantation model and tissue sampling sites. *B*, the mRNA expression levels of *Timeless* in CT2A-derived brain tumors. The mRNA levels were normalized to *18s* rRNA levels. Values in normal brain tissue (sham) were set at 1.0. Each value represents the mean with SD (n = 6). ∗∗*p* < 0.01; significant difference between the two groups (*t*_10_ = 13.855, Student’s *t* test). *C*, TIMELESS protein expression levels in CT2A-derived brain tumor. Protein levels were normalized to β-ACTIN levels. Values in the normal brain tissue (sham) were set at 1.0. Each value represents the mean with SD (n = 6). ∗∗*p* < 0.01; significant difference between the two groups (*t*_10_ = 10.336, Student’s *t* test). *D*, TIMELESS protein expression levels in shScramble and *Timeless* knockdown (KD) CT2A cells. Protein levels were normalized to β-ACTIN levels. Values in shScramble CT2A cells were set at 1.0. Each value represents the mean with SD (n = 3). ∗∗*p* < 0.01; significant difference between the two groups (*t*_4_ = 10.655, Student’s *t* test). *E*, effect of *Timeless-*KD on tumor aggressiveness in an orthotopic CT2A glioblastoma mouse model. shScramble or *Timeless-*KD CT2A cells were intracranially implanted in C57BL/6J mice. Data shows Kaplan-meier survival curves of shScramble or *Timeless-*KD CT2A tumor-bearing mice (n = 7–8). ∗∗*p* < 0.01; significant difference between the two groups (LogRank Holm-Sidak test).

To evaluate the impact of TIMELESS on glioma malignancy *in vivo*, we established an orthotopic transplantation model using C57BL/6J mice by stereotactically implanting CT2A mouse glioma cells into the brain parenchyma (Fig. 2*A*). Consistent with the database analysis of glioma patients, TIMELESS protein and its mRNA levels were higher in CT2A-formed tumor tissues than in normal brain tissues (Fig. 2, *B* and *C*). We then generated CT2A cells with stable *Timeless* knockdown (KD) using a lentivirus expressing *Timeless*-specific shRNA and used a non-targeting scramble shRNA (shScramble) as a control (Fig. 2*D*). After intracranial implantation, mice receiving *Timeless*-KD CT2A cells survived significantly longer than mice receiving shScramble control cells (Fig. 2*E*). These results indicate that TIMELESS contributes to glioma malignancy *in vivo*.

### TIMELESS regulates stemness-associated programs in mouse glioma cells

**Figure 3:**
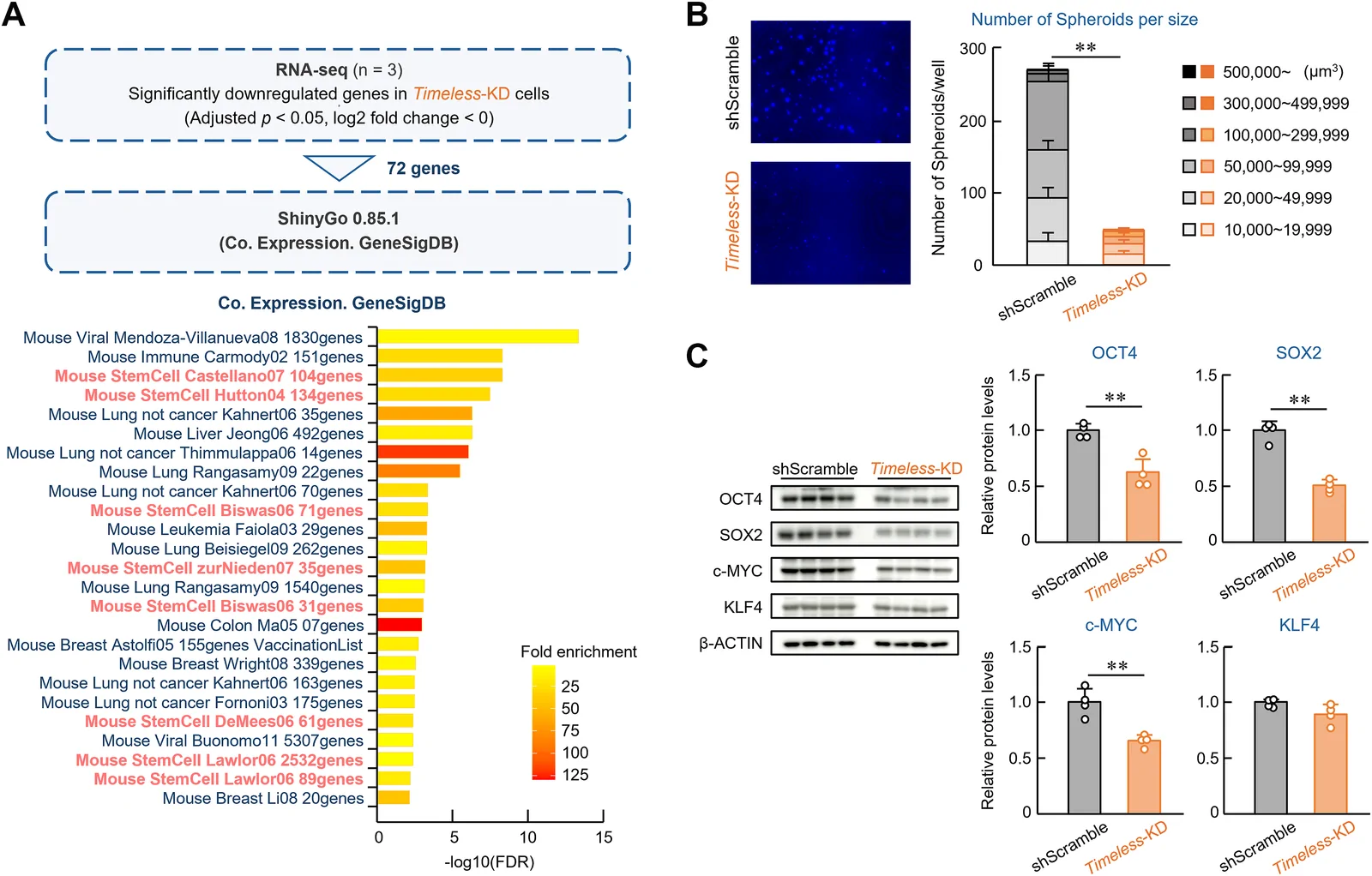
**TIMELESS regulates stemness in mouse glioma CT2A cells.***A*, schematic overview of the strategy used to identify TIMELESS-regulated gene signatures in CT2A cells. The *top panel* shows the workflow for gene selection, and the *bottom panel* shows the results of enrichment analysis based on the Co. Expression. GeneSigDB libraries using ShinyGo v0.85.1 (FDR < 0.01). *B*, spheroid formation ability of shScramble and *Timeless-*knockdown (KD) CT2A cells. The graphs show the number of spheroids and the distribution of spheroid volumes. Values are the mean with SD (n = 7–8). ∗∗*p* < 0.01; significant difference between the two groups (Mann-Whitney *U* test). *C*, protein expression levels of OCT4, SOX2, c-MYC, and KLF4 in shScramble and *Timeless-*KD CT2A cells. Protein levels were normalized to β-ACTIN levels. Values in shScramble-transduced cells were set at 1.0. Each value represents the mean with SD (n = 4). ∗∗*p* < 0.01; significant difference between the two groups (*t*_6_ = 5.387 for OCT4*, t*_6_ = 9.453 for SOX2, *t*_6_ = 5.052 for c-MYC, *t*_6_ = 2.154 for KLF4, Student’s *t* test). OCT4, octamer-binding transcription factor 4; SOX2, sex-determining region Y-box transcription factor 2.

To investigate the mechanisms underlying the *Timeless*-driven glioma malignancy, we performed RNA sequencing (RNA-seq) analysis in *Timeless*-KD and shScramble CT2A cells (Table S1). Among the detected genes, 72 significantly downregulated genes were selected for further analysis (Table S2). We then performed enrichment analysis of this gene set using ShinyGO (v0.85.1), focusing on the Co Expression GeneSigDB libraries. This analysis revealed a significant enrichment of stem cell-related gene signatures (Fig. 3*A*), suggesting that TIMELESS may regulate cancer stem-like properties of glioma cells.

As cancer stem cells can form spheroids under anchorage-independent culture conditions, we assessed spheroid formation using a soft agar assay (21, 22). *Timeless*-KD CT2A cells exhibited markedly reduced spheroid-forming capacity compared with shScramble controls (Fig. 3*B*). Consistently, protein levels of OCT4, SOX2, and c-MYC were significantly decreased in *Timeless*-KD CT2A cells, whereas KLF4 protein levels were largely unchanged (Fig. 3*C*). These findings suggest that TIMELESS selectively promotes stemness-associated features in CT2A cells.

### TIMELESS enhances mouse glioma stemness *via* JAK-STAT signaling

**Figure 4:**
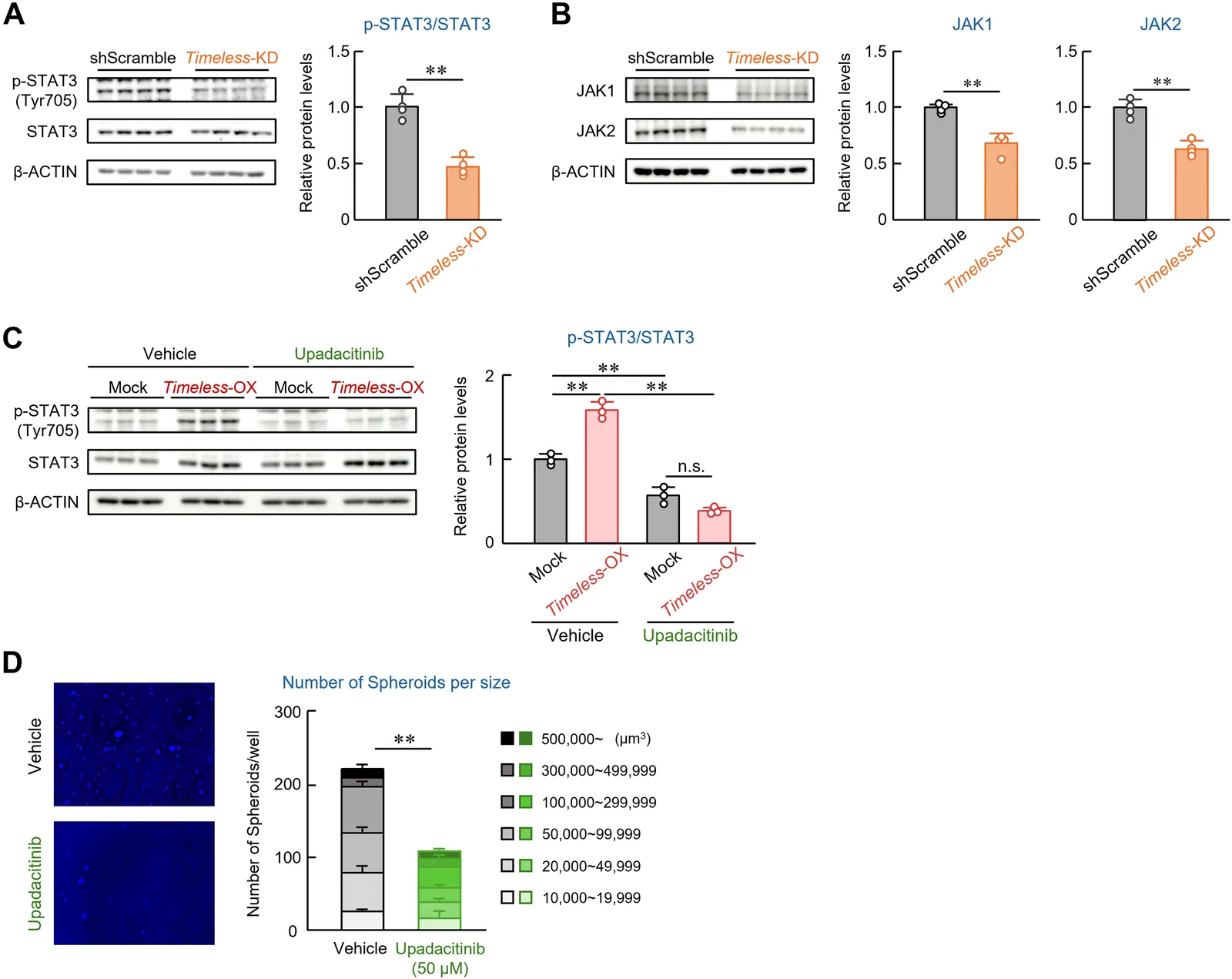
**TIMELESS promotes glioma stemness by enhancing STAT3 phosphorylation through upregulation of JAK expression.***A*, STAT3 phosphorylation states in shScramble and *Timeless-*knockdown (KD) CT2A cells. Phosphorylated STAT3 levels were normalized to total STAT3 expression levels. Values in shScramble-transduced cells were set at 1.0. Each value represents the mean with SD (n = 4). ∗∗*p* < 0.01; significant difference between the two groups (*t*_6_ = 7.572*,* Student’s *t* test). *B*, protein expression levels of JAK1 and JAK2 in shScramble and *Timeless-*KD CT2A cells. The protein levels were normalized to β-ACTIN levels. Values in shScramble-transduced cells were set at 1.0. Each value represents the mean with SD (n = 4). ∗∗*p* < 0.01; significant difference between the two groups (*t*_6_ = 6.624 for JAK1, *t*_6_ = 7.091 for JAK2*,* Student’s *t* test). *C*, STAT3 phosphorylation levels in mock and *Timeless-*overexpressing (OX) CT2A cells at 24 h after treatment with vehicle (0.05% methanol) or upadacitinib (50 μM). Phosphorylated STAT3 levels were normalized to total STAT3 expression levels. Values in mock-transduced cells were set at 1.0. Each value represents the mean with SD (n = 3). ∗∗*p* < 0.01; significant difference between the indicated groups (*F*_3,8_ = 131.427, *p* < 0.001, one-way ANOVA with Tukey–Kramer’s *post hoc* test). *D*, effect of JAK inhibition on spheroid formation in CT2A cells. Photographs show the representative images of spheroid formed by CT2A cells treated with vehicle (0.05% methanol) or upadacitinib (50 μM). *Rightpanel* shows quantification of spheroid number and volume distribution. Values show the mean with SD (n = 4). ∗∗*p* < 0.01; significant difference between the two groups (Mann-Whitney *U* test). STAT3, signal transducer and activator of transcription 3; JAK, janus kinase.

Given that signal transducer and activator of transcription 3 (STAT3) contributes to maintenance of GBM stem-like properties (23, 24, 25), we examined whether TIMELESS regulates STAT3 signaling in CT2A cells. *Timeless* knockdown significantly reduced phosphorylation levels of STAT3 (p-STAT3) in CT2A cells (Fig. 4*A*). As STAT3 phosphorylation is primarily mediated by JAK family members, we also assessed JAK1 and JAK2 protein levels and found that both were decreased in *Timeless*-KD CT2A cells (Fig. 4*B*).

To further examine the involvement of the JAK-STAT3 signaling pathway, we generated CT2A cells stably overexpressing Timeless and evaluated STAT3 phosphorylation. Timeless overexpression significantly upregulated STAT3 phosphorylation compared with mock-transduced cells (Fig. 4*C*). Treatment with upadacitinib, a JAK inhibitor, abolished the Timeless overexpression-induced increase in p-STAT3, reducing p-STAT3 levels to those observed in upadacitinib-treated mock controls (Fig. 4*C*). We next evaluated spheroid formation in soft agar in the presence or absence of upadacitinib. The treatment reduced spheroid formation ability of CT2A cells (Fig. 4*D*). Collectively, these results support a role for JAK-STAT3 signaling in TIMELESS-dependent regulation of stemness-associated properties in CT2A cells.

### TIMELESS enhances stemness-associated properties *via* JAK-STAT3 signaling in human GBM cells

**Figure 5:**
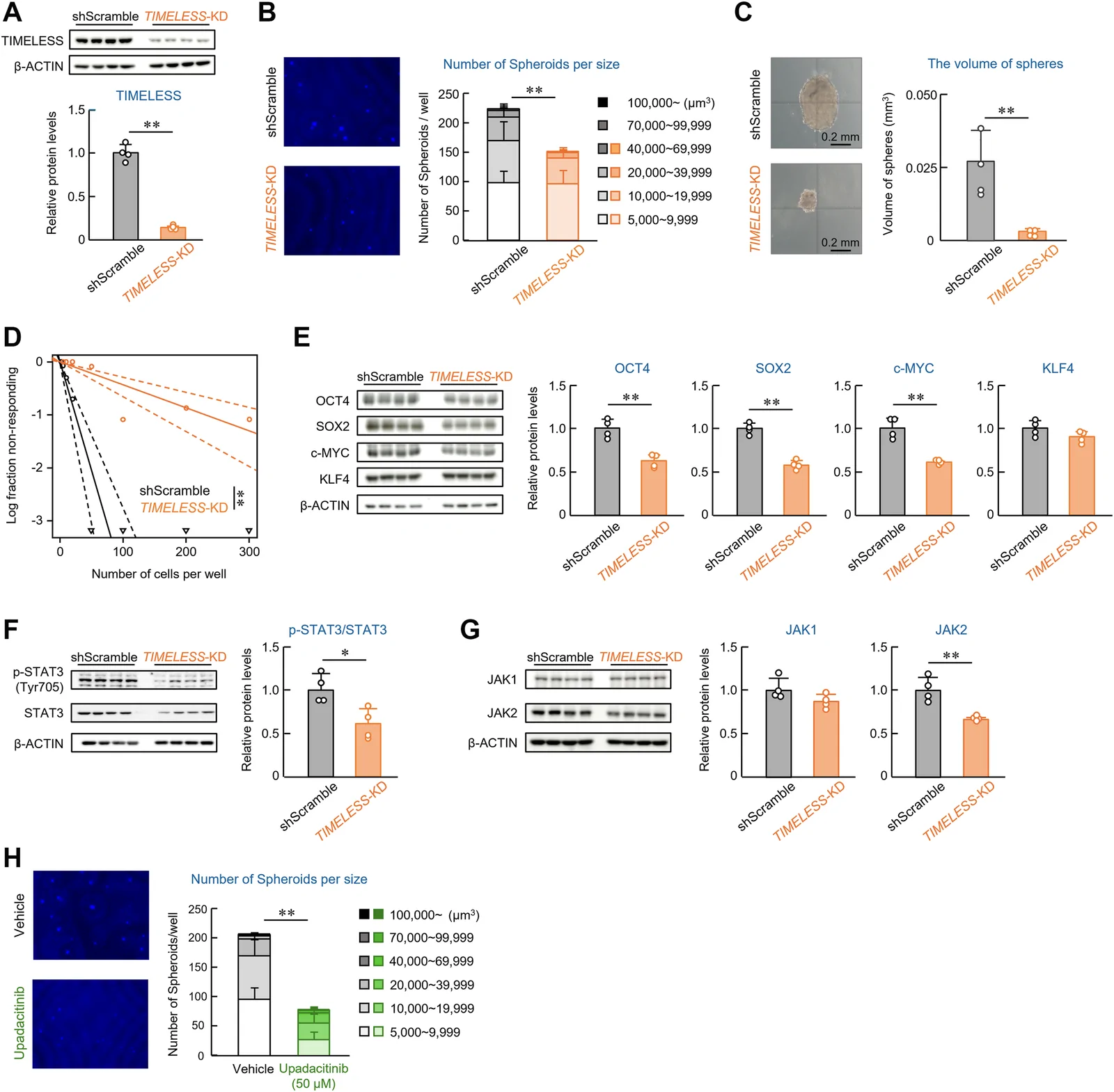
**Knockdown of TIMELESS suppresses glioma stemness in human GBM U251 cells.***A*, TIMELESS protein expression levels in shScramble and *TIMELESS-*knockdown (KD) U251 cells. Protein levels were normalized to β-ACTIN levels. Values in shScramble-transduced cells were set at 1.0. Each value represents the mean with SD (n = 4). ∗∗*p* < 0.01; significant difference between the two groups (*t*_6_ = 17.638, Student’s *t* test). *B*, spheroid formation ability of shScramble and *TIMELESS*-KD U251 cells. *Left photographs* show the representative images of spheroids formed by shScramble or *TIMELESS*-KD U251 cells. *Right panel* shows quantification of spheroid number and volume distribution. Values show the mean with SD (n = 3–4). ∗∗*p* < 0.01; significant difference between the two groups (Mann-Whitney *U* test). *C*, neurosphere formation ability of shScramble and *TIMELESS-*KD U251 cells. *Left photographs* show representative images of neurosphere formed by shScramble or *TIMELESS*-KD U251 cells. *Right panel* shows quantification of neurosphere volume. Values show the mean with SD (n = 4). ∗∗*p* < 0.01; significant difference between the two groups (*t*_6_ = 4.202, Student’s *t* test). *D*, limiting dilution analysis was performed using shScramble and *TIMELESS*-KD U251 cells. Cells were plated at the indicated densities, and wells without sphere formation were counted. The log fraction of negative wells was plotted against the number of cells per well. The *solid line* indicates the estimated stem cell frequency, and the *dotted lines* represent the 95% confidence interval, indicating a significant reduction in stem cell frequency in *TIMELESS*-KD cells. ∗∗*p* < 0.01; significant difference between the two groups. *E*, protein expression levels of OCT4, SOX2, c-MYC, and KLF4 in shScramble and *TIMELESS*-KD U251 cells. Protein levels were normalized to β-ACTIN levels. Values in shScramble U251 cells were set at 1.0. Each value represents the mean with SD (n = 4). ∗∗*p* < 0.01; significant difference between the two groups (*t*_6_ = 5.743 for OCT4*, t*_6_ = 10.042 for SOX2, *t*_6_ = 6.790 for c-MYC, *t*_6_ = 1.802 for KLF4 Student’s *t* test). *F*, STAT3 phosphorylation levels in shScramble and *TIMELESS-*KD U251 cells. Phosphorylated STAT3 levels were normalized to STAT3 expression levels. Values in shScramble-transduced cells were set at 1.0. Each value represents the mean with SD (n = 4). ∗*p* < 0.05; significant difference between the two groups (*t*_6_ = 3.387*,* Student’s *t* test). *G*, protein expression levels of JAK1 and JAK2 in shScramble and *TIMELESS*-KD U251 cells. Protein levels were normalized to those of β-ACTIN levels. Values in shScramble U251 cells were set at 1.0. Each value represents the mean with SD (n = 4). ∗∗*p* < 0.01; significant difference between the two groups (*tGA* = 1.901 for JAK1, *t*_6_ = 4.809 for JAK2*,* Student’s *t* test). *H*, effect of JAK inhibition on spheroid formation in U251 cells. *Left photographs* show the representative images of spheroid formed by U251 cells treated with vehicle (0.05% methanol) or upadacitinib (50 μM). *Rightpanel* shows quantification of spheroid number and volume distribution. Values show the mean with SD (n = 4). ∗∗*p* < 0.01; significant difference between the two groups (Mann-Whitney *U* test). GBM, glioblastoma; OCT4, octamer-binding transcription factor 4; SOX2, sex-determining region Y-box transcription factor 2; JAK, janus kinase.

To determine whether these findings extend to human GBM cells, we generated *TIMELESS*-KD human GBM U251 cells using *TIMELESS*-specific shRNA expressing lentivirus (Fig. 5*A*). Consistent with our observations in CT2A cells, spheroid formation ability was significantly reduced in *TIMELESS*-KD U251 cells compared with shScramble controls (Fig. 5*B*). We also performed a neurosphere formation assay to enrich and expand stem-like cells under serum-free conditions with defined growth factors (26). Neurosphere size was markedly reduced in *TIMELESS*-KD U251 cells compared with shScramble controls (Fig. 5*C*). Furthermore, limiting dilution analysis revealed that the frequency of tumor sphere–initiating cells was markedly reduced in *TIMELESS*-KD cells compared to shScramble cells (1 in 230.7 *versus* 1 in 24.6, respectively), indicating a decrease in the cancer stem cell population in *TIMELESS*-KD U251 cells (Fig. 5*D*).

Consistent with these functional assays, protein levels of OCT4, SOX2 and c-MYC were decreased in *TIMELESS*-KD U251 cells, whereas KLF4 protein levels were largely unchanged (Fig. 5*E*). In addition, phosphorylation of STAT3 was also suppressed in *TIMELESS*-KD U251 cells (Fig. 5*F*). Although JAK2 protein levels were significantly reduced in *TIMELESS* knockdown U251 cells, JAK1 protein levels were not markedly affected (Fig. 5*G*). Finally, upadacitinib treatment suppressed spheroid formation in U251 cells (Fig. 5*H*). These findings suggest that TIMELESS regulates stemness-associated properties in human glioma in association with JAK-STAT3 signaling.

## Discussion

**Figure 6:**
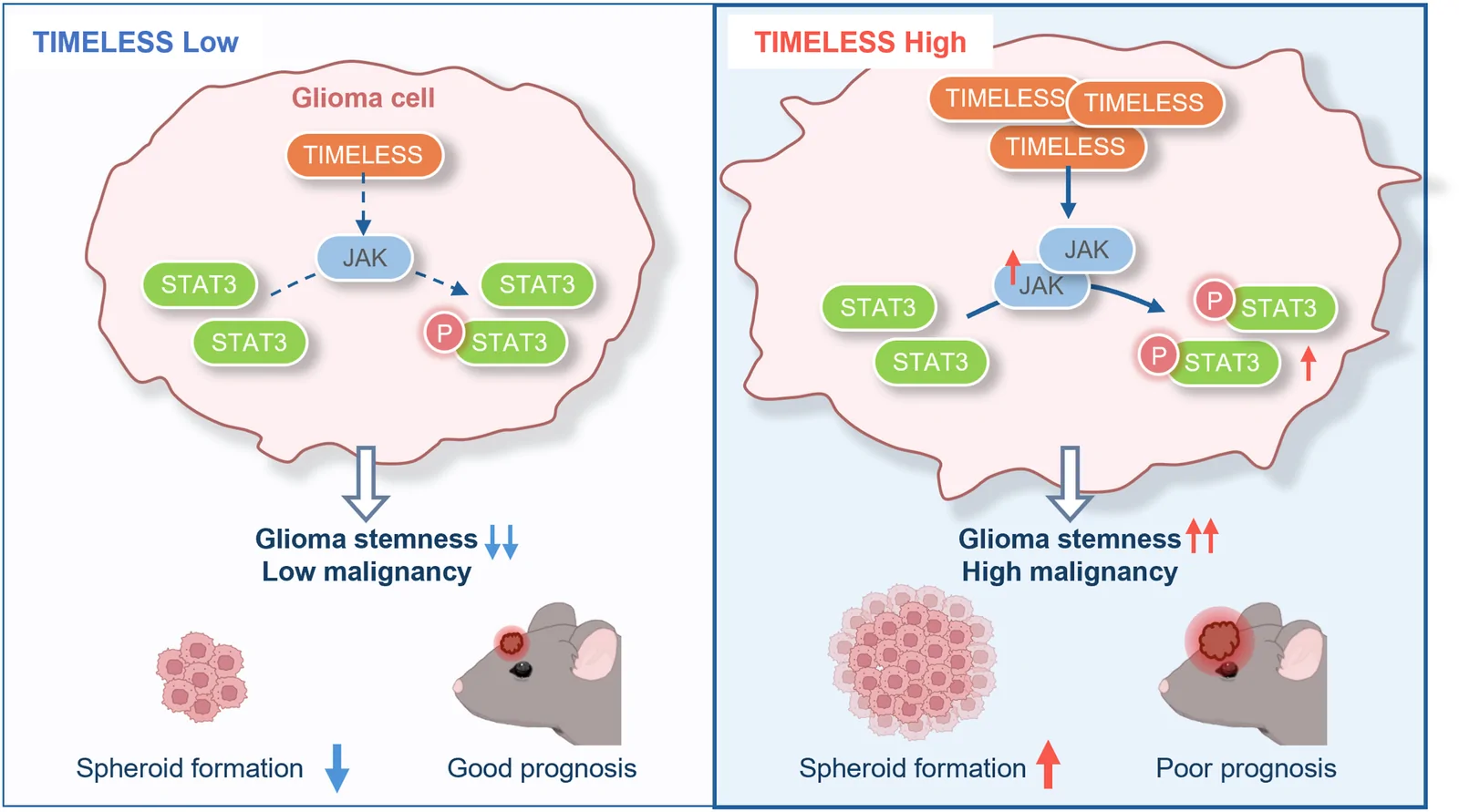
**Schematic diagram of the mechanism by which TIMELESS promotes glioma malignancy.** TIMELESS expression is upregulated in glioma and enhances their malignancy by promoting cancer stemness through activation of JAK-STAT3 signaling pathway. JAK, janus kinase; STAT3, signal transducer and activator of transcription 3.

Glioma stem cells (GSCs) are widely recognized as key drivers of tumor aggressiveness, therapeutic resistance, and recurrence. Rather than reiterating these established features, the present study provides mechanistic insight into how the clock-related protein TIMELESS contributes to glioma stemness by modulating intracellular signaling networks. Our findings support a model in which TIMELESS acts as an upstream regulator of JAK-STAT3 signaling, thereby linking a circadian-related factor to a core pathway sustaining glioma stemness (Fig. 6).

Our analyses demonstrated that *TIMELESS* expression increased with glioma grade and was associated with poor patient prognosis across glioma cohorts. To further assess the prognostic significance of TIMELESS specifically within GBM, we performed Kaplan–Meier survival analyses using GBM-only cohorts from TCGA_GBM, CGGA, Rembrandt, and Gravendeel through GlioVis (Fig. S1). High *TIMELESS* expression was significantly associated with shorter overall survival in the CGGA GBM cohort, whereas this association was not consistently observed across all GBM datasets. One possible explanation is that *TIMELESS* is already highly expressed in GBM (Fig. 1, *A*–*D*, left), which may result in a relatively narrow dynamic range of expression within this subgroup, thereby reducing its prognostic discriminatory power. Together, these findings suggest that TIMELESS is more closely associated with glioma progression than with prognosis within GBM alone.

STAT3 signaling occupies a central role in maintaining GSC self-renewal and tumorigenicity by coordinating transcriptional programs that support pluripotency, survival, and metabolic adaptation (27, 28). In this context, our data suggest that TIMELESS enhances STAT3 activation primarily through upregulation of JAK family kinases, thereby amplifying responsiveness to extracellular stemness-promoting cues. Because JAK–STAT signaling integrates signals from cytokines and growth factors within the tumor microenvironment (29, 30, 31), increased JAK expression driven by TIMELESS may act as a signal-amplifying mechanism that reinforces stemness-associated transcriptional programs under permissive conditions.

An important aspect of our study is the selective regulation of stemness-associated transcription factors downstream of TIMELESS. Although OCT4, SOX2, c-MYC, and KLF4 are frequently grouped as core stemness regulators, their functional hierarchies differ across tumor types and signaling contexts (5). In glioma, OCT4, SOX2, and c-MYC are closely linked to STAT3 activity and contribute directly to the maintenance of stemness and tumorigenicity (27, 28). In contrast, KLF4 has been reported to exert context-dependent, and in some cases tumor-suppressive, functions in multiple cancers (32, 33). Notably, KLF4 expression was upregulated following STAT3 inhibition in glioma cells treated with the STAT3 inhibitor STX-0119 (27), further supporting a functional distinction between KLF4 and other STAT3-responsive stemness factors. Consistent with these observations, TIMELESS knockdown in both mouse glioma and human GBM cells selectively reduced OCT4, SOX2, and c-MYC protein expression, while leaving KLF4 levels largely unchanged. Together, these findings support a model in which TIMELESS selectively reinforces a STAT3-centered stemness axis rather than globally activating pluripotency-associated transcriptional networks.

Beyond JAK-STAT3 signaling, TIMELESS has been shown to interact with transcription factors, including specificity protein 1 (SP1) and JUN, a core component of the activator protein-1 (AP-1) complex, and to regulate the expression of downstream target genes and microRNAs (34, 35). These interactions suggest that TIMELESS functions as a hub molecule integrating transcriptional control capable of coordinating multiple oncogenic pathways. Consistent with this notion, enrichment of SP1 and AP-1 binding motifs in the regulatory regions of *JAK* genes supports a transcriptional mechanism by which TIMELESS may directly or indirectly control JAK expression. Although this study focused on JAK-STAT3 signaling, TIMELESS may also influence additional GSC-associated programs, including metabolic and epigenetic regulation.

We observed species-dependent differences in JAK regulation downstream of TIMELESS, with suppression of both JAK1 and JAK2 in mouse CT2A cells, but selective downregulation of JAK2 in human U251 cells. Computational analysis of upstream regulatory regions of *JAK1* gene using JASPAR database (https://jaspar.elixir.no/) suggests that these differences may reflect divergence in transcriptional and posttranscriptional regulatory architectures between mouse and human JAK genes, highlighting the need for careful interpretation when extrapolating mechanistic findings from murine models to human glioma biology.

Although *Timeless* was originally identified as a circadian-related protein (36)*,* our data indicate that its role in glioma stemness is largely independent of overt alterations in core circadian gene expression. Indeed, *Timeless* knockdown did not significantly affect the expression of major circadian clock genes, including *Clock*, *Bmal1*, *Per2*, and *Nr1d1* (Fig. S2*A*). Furthermore, we evaluated the clock-independent function of TIMELESS using the functional mutant R1081X TIMELESS (a truncation mutant lacking the C-terminal region downstream of Arg1081), which has been reported to be defective in canonical circadian regulation (37). R1081X TIMELESS overexpression enhanced STAT3 activation and spheroid forming ability in U251 cells, comparable to wild-type TIMELESS overexpression (Fig. S2, *B*–*D*). This dissociation supports the emerging concept that certain clock-related proteins can be repurposed in cancer cells to regulate oncogenic signaling networks without directly perturbing canonical circadian oscillators. Given that core clock genes such as BMAL1 and CLOCK promote glioma stemness and tumor aggressiveness (15), TIMELESS may act in parallel with, rather than upstream of, the core circadian transcriptional machinery.

TIMELESS has been implicated in the maintenance of genomic stability and the regulation of cell cycle checkpoints (38, 39, 40). To determine whether the observed phenotypes are associated with alterations in genomic stability, we assessed γH2AX levels as an indicator of DNA damage accumulation. However, TIMELESS depletion did not alter γH2AX levels (Fig. S3), suggesting that the effects of TIMELESS on glioma cells are unlikely to be mediated by changes in DNA damage under our experimental conditions. In contrast, *TIMELESS* knockdown induced G_2_/M arrest in CT2A cells and G_0_/G_1_ arrest in U251 cells (Fig. S4), indicating that TIMELESS contributes to cell cycle regulation in glioma cells. Notably, the extent of cell cycle alteration in U251 cells by TIMELESS was relatively limited, whereas stemness-associated phenotypes, including impaired sphere-forming ability and reduced expression of stem cell markers, were consistently observed following TIMELESS depletion. These findings suggest that the attenuation of glioma stem-like properties cannot be fully explained by secondary effects resulting from cell cycle perturbation. Rather, TIMELESS may play a more direct role in maintaining glioma stemness.

Finally, analysis of patient-derived transcriptomic datasets identified Enhancer of zeste homolog 2 (EZH2) as a gene strongly correlated with TIMELESS expression across multiple glioma cohorts (CGGA, TCGA_GBM, Rembrandt, and Gravendeel; Fig. S5). EZH2 is frequently overexpressed in glioma and plays critical roles in stem cell maintenance, tumor invasiveness, and poor prognosis (41, 42). In addition to its canonical role as a histone methyltransferase, EZH2 can function as a transcriptional coregulator independent of its enzymatic activity (42, 43). Based on these findings, EZH2 may contribute to the upregulation of *TIMELESS* by stabilizing transcription factor binding, recruiting transcriptional coactivators, thereby linking epigenetic regulation to TIMELESS-driven stemness.

In summary, this study identifies TIMELESS as a previously unrecognized regulator of glioma stemness that operates through JAK-STAT3 signaling. By connecting a circadian-associated factor to a central stemness pathway, our findings expand the functional repertoire of TIMELESS and suggest that targeting the TIMELESS-JAK-STAT3 axis may represent a promising strategy to disrupt stemness-driven malignancy in glioma.

## Experimental procedures

### Glioma patients Kaplan-Meier survival analyses

*TIMELESS* mRNA expression levels and their association with patient survival were analyzed using datasets of the CGGA, TCGA_GBMLGG, Rembrandt and Gravendeel *via* GlioVis, a data portal for visualization and analysis of brain tumor transcriptome datasets (http://gliovis.bioinfo.cnio.es) (19). For each dataset, TIMELESS expression levels were compared across glioma grades, and overall survival was evaluated using Kaplan–Meier analysis with patients stratified into high- and low-expression groups based on the median expression value.

### Animals and treatments

All protocols using mice were reviewed and approved by the Animal Care and Use Committee of Kyushu University. All methods were performed in accordance with the relevant guidelines and regulations. Male C57BL/6J mice were purchased from Jackson Laboratory Japan (Yokohama). Mice were housed in groups (from six to eight per cage) in a light-controlled room (lights on 7:00 AM–7:00 PM) at 24 ± 1 °C, with humidity at 60 ± 10%, and provided with food and water *ad libitum*. Orthotopic CT2A glioblastoma model mice were established using the method described previously (44). Briefly, CT2A cells (1.0 × 10^5^ cells) were suspended in 5 μl of PBS and implanted slowly over 5 min into the brain of 6 weeks old male C57BL/6J mice anesthetized with isoflurane inhalation anesthesia (Pfizer).

### Cell and treatment

CT2A mouse glioma cells were purchased from Sigma-Aldrich (St Louis, MO, RRID: CVCL_ZJ44). U251 human glioblastoma cells were purchased from the National Institute of Biomedical Innovation (Osaka, Japan, RRID: CVCL_0021). Lenti-X 293T cells were purchased from Takara Bio (Cat# 632180). CT2A cells were cultured in high-glucose Dulbecco’s modified Eagle’s medium (DMEM; Fujifilm Wako Pure Chemical Co) supplemented with 10% Fetal Bovine Serum (FBS; Biowest) and 0.25% penicillin-streptomycin solution (Fujifilm Wako Pure Chemical). U251 and Lenti-X 293T cells were cultured in DMEM (Thermo Fisher Scientific) supplemented with 10% FBS and 0.25% penicillin-streptomycin solution (Fujifilm Wako Pure Chemical). All cell lines were confirmed to be free of *mycoplasma* contamination using the MycoBlue *Mycoplasma* Detector (Vazyme Biotech). Cells were maintained at 37 °C in a humidified 5% CO_2_ atmosphere. For each experiment, cells were seeded onto culture plates and allowed to adhere for 24 h. Subsequently, upadacitinib (Cat# 29706; Cayman Chemical) was added to the culture medium at the indicated concentrations, and cells were incubated for an additional 24 h. Control cells were treated with an equivalent volume of methanol, which was used as the solvent for upadacitinib.

### Quantitative real-time RT-PCR analysis

Total RNA was extracted from tissues using RNAiso (Takara Bio) according to the manufacturer’s instructions. The extracted total RNA was reverse transcribed using the ReverTra Ace qPCR Kit (Toyobo, Osaka, Japan) to synthesize cDNA samples. Using this obtained cDNA as a template, PCR reactions were performed using THUNDERBIRD SYBR qPCR mix (Toyobo) and the LightCycler 96 System (Roche Diagnostics). Gene expression levels were quantified using the primers listed in Table 1. 18S ribosomal RNA was used as an internal standard.

**Table 1:** Primer sets for RT-PCR analysis

Gene	Primer sequence		Species
*Timeless*	Forward	5′- ATGAACTGTGAACTTCTAGCCAC -3′	Mouse
	Reverse	5′- CCTCAGGTATCGGATCAAATCCT -3′	
*Clock*	Forward	5′- TTGCTCCACGGGAATCCTT -3′	Mouse
	Reverse	5′- GGAGGGAAAGTGCTCTGTTGTAG -3′	
*Bmal1*	Forward	5′- GGACTTCGCCTCTACCTGTTCA -3′	Mouse
	Reverse	5′- AACCATGTGCGAGTGCAGGCGC -3′	
*Period2*	Forward	5′- GACTGCGACGACAATGGGAA -3′	Mouse
	Reverse	5′- TTTGGCAGACTGCTCACTACT -3′	
*Nr1d1*	Forward	5′- CCCTGGACTCCAATAACAACACA -3′	Mouse
	Reverse	5′- GCCATTGGAGCTGTCACTGTAG -3′	
*18S* rRNA	Forward	5′- CGGCTACCACATCCAAGGAA -3′	Mouse/Human
	Reverse	5′- GCTGGAATTACCGCGGCT -3′	

### Western blotting

For total protein extraction, cells and tissues were lysed with radio-immunoprecipitation assay (RIPA) buffer (20 mM Tris-HCl (pH 7.4), 150 mM NaCl, 0.1% SDS, 1% NP-40, 0.5% Deoxycholic acid, 2 mM EDTA). The extracts were centrifuged at 15,000×*g* for 10 min at 4 °C, and the supernatant was collected. The protein extracts were mixed with an equal volume of 2×sample buffer (250 mM Tris (pH 6.8), 2% SDS, 30% (v/v) Glycerol, 0.01% Bromophenol blue, 10% (v/v) 2-mercaptoethanol) and denatured at 95 °C for 5 min.

The samples were separated by sodium dodecyl sulfate-polyacrylamide gel electrophoresis (SDS-PAGE) and transferred onto a polyvinylidene difluoride membrane (Millipore). DynaMarker protein multicolor ladder marker stable II (BioDynamics Laboratory) was used as a protein ladder marker. The membranes were incubated with antibodies against TIMELESS (Cat# 67022-1-Ig, Proteintech, RRID: AB_2882337), OCT4 (Cat# 653701, Biolegend, RRID: AB_2561766), SOX2 (Cat# AF2018, R&D systems, RRID: AB_355110), c-MYC (Cat# 10828-1-AP, Proteintech, RRID: AB_2148585), KLF4 (Cat# 11880-1-AP, Proteintech, RRID: AB_10640807), STAT3 (Cat# 678001, Biolegend, RRID: AB_2861053), STAT3 Phospho (Tyr705) (Cat# 651001, Biolegend, RRID: AB_10897947), JAK1 (Cat# sc-1677, Santa Cruz Biotechnology, RRID: AB_627836), JAK2 (Cat# F0231, Selleck Chemicals), p84 (THOC1, Cat# 10920-1-AP, Proteintech, RRID: AB_2202239), GAPDH (Cat# 5174S, Cell Signaling Technology, RRID: AB_10622025), gamma H2A.X Phospho (S139) (Cat# MABI0281–20, Monoclonal Antibody Research Institute Inc.) or anti-β-ACTIN antibody conjugated with horseradish peroxidase (Cat# sc-47778, Santa Cruz Biotechnology, RRID: AB_2202239). Specific antigen–antibody complexes were visualized using HRP-conjugated anti-rabbit IgG antibody (Cat# ab97051, Abcam, RRID: AB_10679369), HRP-conjugated anti-goat IgG antibody (Cat# sc-2020, Santa Cruz Biotechnology, RRID: AB_631728), HRP-conjugated anti-mouse IgG antibody (Cat# ab6820, Abcam, RRID: AB_955438) and ImmunoStar LD (Fujifilm Wako Pure Chemical). Blotting images were scanned using LAS-3000 (Fuji Film Co.) or ImageQuant LAS4010 (GE Healthcare Life Sciences). The band intensity of western blotting was quantified using Image J (version 1.8.0, NIH, RRID: SCR_003070). The obtained data were normalized to the housekeeping protein, β-ACTIN.

### Construction of *Timeless* knockdown or overexpression CT2A and U251 cells

Scramble shRNA (Scramble[shRNA#1]), mouse *Timeless* shRNA (pLV[shRNA]-Puro-U6>mTimeless[shRNA#1]), mouse *Timeless* (pLV[Exp]-Puro-CMV>mTimeless) and human *TIMELESS* shRNA (pLV[shRNA]-Puro-U6>hTimeless[shRNA#1]) expressing plasmid were purchased from VectorBuilder. The sequences of the shRNAs were listed in Table 2. Lentiviral particles were prepared using the Lentiviral High Titer Packaging Mix with pLVSIN series (TaKaRa Bio Inc.) according to the manufacturer's protocol. Lenti-X 293T cells were used for lentivirus packaging. These lentivirus particles were used to transduce CT2A and U251 cells in the presence of 10 μg/ml polybrene (Sigma-Aldrich). Transduced cells were selected by culturing in growth medium containing 10 μg/ml of puromycin (Fujifilm Wako Pure Chemical).

**Table 2:** Sequences of the shRNAs

Target gene	Sequence	Species
*Timeless*	5′- TGATCAGGCTGATGGTAAATTCTC GAGAATTTACCATCAGCCTGATCA -3′	Mouse
*TIMELESS*	5′- GCCGCATCATCAAGAACAATACTC GAGTATTGTTCTTGATGATGCGGC -3′	Human

### RNA-seq analysis

Total RNA was extracted from three independent biological replicates of shScramble and *Timeless-KD* CT2A cells using the FastGene RNA Basic Kit with DNaseI (Cat# FG-80250, Nippon Genetics). Bulk RNA barcoding and sequencing (BRB-seq) libraries were prepared as previously described with minor modifications (45). Briefly, first-strand cDNA synthesis was performed using an oligo(dT)-based primer, followed by second-strand synthesis using the Second Strand Synthesis Module (New England Biolabs, Ipswich). Double-stranded cDNA was subjected to tagmentation using an in-house MEDS-B Tn5 transposase (46, 47), and the libraries were amplified by 10 cycles of PCR using Phusion High-Fidelity DNA Polymerase (Thermo Fisher Scientific). Sequencing was performed on the Illumina NextSeq 2000 platform to generate 81-bp paired-end reads. For data processing, sample barcodes and unique molecular identifiers (UMIs) were extracted using UMI-tools (v1.1.6). Adapter sequences and low-quality bases were removed using Trim Galore (v0.6.11), and reads shorter than 20 bp after trimming were discarded. The processed reads were aligned to the mouse reference genome (GRCm38) using HISAT2 (v2.2.2). Gene-level read counts were generated using featureCounts (v2.1.1). Differential expression analysis was performed in R (v4.5.3) using DESeq2 (v1.50.2). Prior to analysis, genes with at least 10 read counts in at least three samples were retained for downstream analysis. Genes significantly downregulated in *Timeless*-KD cells compared with shScramble control cells (adjusted *p* < 0.05 and log2 fold change < 0) were subjected to enrichment analysis using the Co-expression Gene Signature database (GeneSigDB) through ShinyGO (v0.85.1) (https://bioinformatics.sdstate.edu/go/).

### Spheroid formation assay

To assess the growth ability of cells in an anchorage-independent manner, the cells were cultured under nonadherent conditions. The bottom of a 24-well plate was coated with soft agarose medium (Growth medium containing 0.75% SeaPlaque GTG Agarose (Lonza)), then cooled at 4 °C for 8 min to solidify. CT2A and U251 cells were suspended in top soft agarose medium (Growth medium containing 0.36% SeaPlaque GTG Agarose) and were seeded at a density of 4 × 10^3^ cells per well onto agarose medium coated well. The cells were cooled at 4 °C for 8 min to solidify and were cultured for 10 days. Upadacitinib solution (Cayman Chemical) was mixed with top soft agarose medium (0.36% agarose). As vehicle control, methanol at the same concentration as upadacitinib solution was mixed with top soft agarose medium (0.36% agarose). The formed-spheroids were stained with Hoechst 33342 (DOJINDO LABORATORIES) dissolved to a final concentration of 2 μg/ml and incubating for 30 min and were photographed using the KEYENCE all-in-one microscope BZ-X800 (KEYENCE). The number and size of spheroids were quantified by the BZ Analyzer software (KEYENCE).

### Neurosphere formation assay

The bottom of a 24-well plate was coated with Polyhema (Poly(2-hydroxyethyl methacrylate), Cat# P3932, Sigma-Aldrich). U251 cells were suspended in neurosphere medium (DMEM supplemented with 20 ng/ml recombinant human epidermal growth factor (EGF; Cat# AF-100–15, Peprotech), 20 ng/ml recombinant basic fibroblast growth factor (bFGF; Cat# 234-FSE, R&D systems) and 10 ng/ml recombinant human leukemia inhibitory factor (LIF; Cat# 593902, Biolegend), B-27 Supplement (Cat# 17504044, Thermo Fisher Scientific), and 0.25% penicillin-streptomycin solution (Fujifilm Wako Pure Chemical), seeded at a density of 1 × 10^4^ cells per well and cultured for 30 days. Neurospheres were photographed using phase-contrast microscope and sizes were measured using ImageJ software (version 1.8.0, NIH).

### Limiting dilution analysis

U251 cells were suspended in neurosphere medium described previously. Cells were seeded at a density of 2, 5, 10, 20, 50, 100, 200 and 300 cells per well into a Prime Surface 96-U-plate (Cat# MS-9096U, Sumitomo Bakelite), with 12 wells per condition, and cultured for 10 days. The proportion of wells without spheres was calculated for each cell density and plotted against the number of seeded cells. Stem cell frequency was estimated using the Extreme Limiting Dilution Analysis (ELDA) online software (http://bioinf.wehi.edu.au/software/elda/).

### Cytoplasmic and nuclear fractionation

Cytoplasmic and Nuclear fractions were extracted using LysoPure Nuclear and Cytoplasmic Extractor Kit (Cat# 295–73901, Fujifilm Wako Pure Chemical) according to the manufacturer’s instructions. The nuclear fraction was lysed in SDS lysis buffer and sonicated to shear genomic DNA.

### Histone extraction

The histone extraction procedure was previously described (48). Briefly, cells were lysed with ice-cold histone extraction buffer, incubated on ice for 10 min, and centrifuged at 13,000*g* at 4 °C for 1 min. The obtained pellet was resuspended in 0.2 M HCl. After vortex mixing, it was centrifuged at 13,000*g* at 4 °C for 20 min. The supernatant was transferred to a new tube, and proteins were precipitated by addition of 100% trichloroacetic acid. After vortex mixing, it was centrifuged at 13,000*g* at 4 °C for 20 min. The pellet was washed with 500 μl of acetone. After vortex mixing, it was centrifuged at 13,000*g* at 4 °C for 2 min. The acetone was discarded, and the pellet was air-dried at RT. Finally, the pellet was resuspended in sample buffer and boiled at 95 °C for 5 min.

### Cell cycle analysis

Cells were harvested by trypsinization. Obtained cells were fixed in 70% ethanol for 1 h at 4 °C and washed with ice-cold hank’s balanced salt solution (HBSS) containing 1% FBS. The cells were resuspended in 1 ml of PBS containing 0.5 mg/ml ribonuclease (Cat# R4875, Sigma-Aldrich) and incubated at 37 °C for 1 h. Then, the cells were washed and incubated with 500 μl of PBS containing 50 μg/ml of propidium iodide (PI; Cat# 169–26281, Fujifilm Wako Pure Chemical) at 4 °C for 2 h. Cell population analysis was performed using BD FACSLyric system (BD Biosciences). Data were processed using FlowJo version 10.10.0 software (BD Biosciences).

### Statistical and data analyses

All statistical analyses were conducted using JMP pro 17 software (SAS Institute). Results are expressed as mean with SD. All data were checked for normality and equal variances before performing ANOVA. The comparison of multiple groups was evaluated using one-way ANOVA followed by Tukey-Kramer *post hoc* test, or Kruskal-Wallis test with Steel-Dwass test. The comparison of two groups was analyzed using either unpaired two-tailed Student’s *t* test or Mann–Whitney *U* test. The comparison of Kaplan-Meier survival curves data was assessed by LogRank Holm-Sidak test. It was considered significant if the *p* value was < 0.05.

## Data availability

All data supporting the results of the present study are included in the article.

The RNA-seq data generated in this study have been deposited in the Gene Expression Omnibus (GEO) database under accession number GSE336084.

## Supporting information

This article contains supporting information.

## Conflict of interest

The authors declare that they have no conflicts of interest with the contents of this article.

## Supporting information

Supplementary Figure1
