# Supplementary material for: TIMELESS promotes glioma stemness and malignancy through JAK-STAT3 pathway
Source: J Biol Chem. 2026 Aug 13;302(10):113447. doi: 10.1016/j.jbc.2026.113447 (PMC13579946; doi:10.1016/j.jbc.2026.113447)
Supplement: Supplementary file 1 [file mmc1.docx]

**TIMELESS promotes glioma stemness and malignancy through JAK-STAT3 pathway**

**Running title: TIMELESS drives glioma malignancy**

Yuichiro Kai^1,^^‡^, Akito Tsuruta^1,‡,^*, Takuto Inoki^1^, Tomoaki Yamauchi^1^, Shigehiro Ohdo^2^, and Satoru Koyanagi^1,^*.

^‡^ These authors contributed equally to this work.

* For correspondence: Akito Tsuruta; Satoru Koyanagi.

**Supplementary Figure S1** Kaplan-Meier survival analysis of GBM-only cohorts stratified by TIMELESS expression.

**Supplementary Figure S2** The influence of TIMELESS on the other clock genes in glioma cells.

**Supplementary Figure S3** The influence of *TIMELESS*-KD on the genomic stability in CT2A and U251 cells.

**Supplementary Figure S4** The influence of *TIMELESS*-KD on cell cycle in CT2A and U251 cells.

**Supplementary Figure S5** Exploration of potential regulatory genes for TIMELESS expression.

**Supplementary Figure S6** Unedited full blots of Figure 2C

**Supplementary Figure S7** Unedited full blots of Figure 2D

**Supplementary Figure S8** Unedited full blots of Figure 3C

**Supplementary Figure S9** Unedited full blots of Figure 4A

**Supplementary Figure S10** Unedited full blots of Figure 4B

**Supplementary Figure S11** Unedited full blots of Figure 4C

**Supplementary Figure S12** Unedited full blots of Figure 5A

**Supplementary Figure S13** Unedited full blots of Figure 5E

**Supplementary Figure S14** Unedited full blots of Figure 5F

**Supplementary Figure S15** Unedited full blots of Figure 5G

**Supplementary Figure S16** Unedited full blots of Figure S2B

**Supplementary Figure S17** Unedited full blots of Figure S2C

**Supplementary Figure S18** Unedited images of Figure S3A

**Supplementary Figure S19** Unedited images of Figure S3B

**Supplementary Figure S1** **Kaplan-Meier survival analysis of GBM-only cohorts stratified by *TIMELESS* expression.**

Kaplan–Meier survival analyses were performed to evaluate the association between *TIMELESS* mRNA expression and overall survival in patients with GBM. GBM patient data were obtained from the TCGA_GBM **(A)**, CGGA **(B)**, Rembrandt **(C),** and Gravendeel **(D)** databases and analyzed using GlioVis. Patients in each cohort were stratified into *TIMELESS*-high and *TIMELESS*-low expression groups using the median expression level as the cutoff. **p* < 0.05; significant difference between the high- and low-expression groups (LogRank Holm-Sidak test).

**Supplementary Figure S2 The influence of TIMELESS on the other clock genes in glioma cells.**

**(A)** The mRNA expression levels of *Clock*, *Bmal1*, *Period2 (Per2)*, and *Nr1d1* in shScramble and *Timeless* knockdown (KD) CT2A cells. Values of mRNA levels were normalized to those of *18s* rRNA levels. Values show the mean with SD (n = 5). (*t*_8_ = 0.909 for *Clock*, *t*_8_ = 0.873 for *Bmal1, t*_8_ = 2.083 for *Per2, t*_8_ = 1.227 for *Nr1d1,* Student’s *t*-test). **(B)** Protein expression levels of TIMELESS in the cytoplasm or nuclei of *TIMELESS* (WT) overexpressing (OX) and *TIMELESS* (R1081X) OX U251 cells. The protein levels were normalized to those in cytoplasm levels. Values in *TIMELESS WT-*transduced cells were set at 1.0. Each value represents the mean with SD (n = 3). ***p* < 0.01; significant difference between the two groups (*t*_4_ = 20.322, Student’s *t*-test). **(C)** STAT3 phosphorylation states in Mock, *TIMELESS* (WT) OX, and *TIMELESS* (R1081X) OX U251 cells. Phosphorylated STAT3 levels were normalized to total STAT3 expression levels. Values in mock-transduced cells were set at 1.0. Each value represents the mean with SD (n = 3). **p* < 0.05, ***p* < 0.01; significant difference between the two groups (*F*_2,6_ = 27.954, *p* < 0.001, one-way ANOVA with Tukey–Kramer’s post hoc test). **(D)** Spheroid formation ability of Mock, *TIMELESS* (WT) OX, and *TIMELESS* (R1081X) OX U251 cells. Top photographs show the representative images of spheroids formed by Mock, *TIMELESS* (WT) OX, and *TIMELESS* (R1081X) OX U251 cells. Bottom panel shows quantification of spheroid number and diameter distribution. Values show the mean with SD (n = 4). ***p* < 0.01; significant difference between the two groups (Kruskal-Wallis test with Steel-Dwass test).

**Supplementary Figure S3 The influence of *TIMELESS*-KD on the genomic stability in CT2A and U251 cells.**

**(A, B)** Phosphorylation of serine 139 residue in histone H2AX (γH2AX) in shScramble and *Timeless*-KD CT2A cells **(A)** and shScramble and *TIMELESS*-KD U251 cells **(B)**. Left panel shows membrane image of western blot analysis using anti-γH2AX antibodies and gel image of CBB staining in extracted histone sample. Right panel shows quantified γH2AX levels. The γH2AX levels were normalized to that of H2A levels. The values in shScramble cells were set at 1.0. Each value represents the mean with SD (n = 3).

**Supplementary Figure S4 The influence of *TIMELESS*-KD on cell cycle in CT2A and U251 cells.**

**(A, B)** Cell cycle distribution in shScramble and *Timeless*-KD CT2A cells **(A)**, shScramble and *TIMELESS*-KD U251 cells **(B)**. Left panel shows representative histograms, and right panel shows quantification of the cell cycle distribution. Values show the mean with SD (n = 5). **p* < 0.05, ***p* < 0.01; significant difference between the two groups. (for panel A, *t*_8_ = 3.752 (G_0_/G_1_ phase)*, t*_8_ = 7.347 (S phase), *t*_8_ = 8.403 (G_2_/M phase), for panel B, *t*_8_ = 3.244 (G_0_/G_1_ phase)*, t*_8_ = 2.484 (S phase), *t*_8_ = 1.189 (G_2_/M phase), Student’s *t*-test).

**Supplementary Figure S5 Exploration of potential regulatory genes for TIMELESS expression.**

Schematic diagram illustrating the identification of potential regulatory genes for TIMELESS expression using GlioVis. The upper panel shows the workflow for gene selection, and the lower scatter plot shows *TIMELESS* mRNA and *EZH2* mRNA expression levels. Blue line indicates regression line.

**Supplementary Figure S6 Unedited full blots of Figure 2C**

**Supplementary Figure S7 Unedited full blots of Figure 2D**

**Supplementary Figure S8 Unedited full blots of Figure 3C**

**Supplementary Figure S9 Unedited full blots of Figure 4A**

**Supplementary Figure S10 Unedited full blots of Figure 4B**

**Supplementary Figure S11 Unedited full blots of Figure 4C**

**Supplementary Figure S12 Unedited full blots of Figure 5A**

**Supplementary Figure S13 Unedited full blots of Figure 5E**

**Supplementary Figure S14 Unedited full blots of Figure 5F**

**Supplementary Figure S15 Unedited full blots of Figure 5G**

**Supplementary Figure S16 Unedited full blots of Figure S2B**

**Supplementary Figure S17 Unedited full blots of Figure S2C**

**Supplementary Figure S18 Unedited images of Figure S3A**

**Supplementary Figure S19 Unedited images of Figure S3B**
